# An engineered TIMP2-based and enediyne-integrated fusion protein for targeting MMP-14 shows potent antitumor efficacy

**DOI:** 10.18632/oncotarget.4709

**Published:** 2015-07-20

**Authors:** Jian Xu, Xiu-Jun Liu, Liang Li, Sheng-Hua Zhang, Yi Li, Rui-Juan Gao, Yong-Su Zhen

**Affiliations:** ^1^ Institute of Medicinal Biotechnology, Chinese Academy of Medical Sciences & Peking Union Medical College, Beijing, China

**Keywords:** MMP-14, TIMP2, fusion protein, targeted therapeutics, antitumor efficacy

## Abstract

Recent studies have shown that MMP-14 is highly expressed in a panel of human solid tumors and poses as a potential molecular target for anticancer drugs. Currently, major strategies for targeted therapeutics have mainly focused on the use of antibody or ligand-based agents. For seeking an alternative approach, it is of interest to employ endogenous proteins as drug delivery carriers. Considering the facts that TIMP2, the tissue inhibitor of metalloproteinase 2, shows specific interaction with MMP-14 and that Lidamycin (LDM), an extremely potent cytotoxic antitumor antibiotic, consists of an apoprotein (LDP) and a highly active enediyne (AE); we designed and prepared a TIMP2-based and enediyne-integrated fusion protein LDP(AE)-TIMP2 by DNA recombination and molecular reconstitution consecutively. Furthermore, the MMP-14 binding attributes of the active fusion protein were determined and its therapeutic efficacy against human esophageal carcinoma KYSE150 xenograft and human fibrosarcoma HT1080 xenograft models in nude mice was investigated. It is suggested that TIMP2, the endogenous and MMP-14 binding protein, might serve as a guided carrier for targeted therapeutics.

## INTRODUCTION

The development of targeted therapeutics, for instance, antibody-based drugs, has made great progress in recent years [1]. Besides antibody-based drugs, exploring and developing new approaches, such as, the use of endogenous proteins for targeted cancer therapy are attractive and urgently needed. In particular, it is essential to seek or design more efficient carriers for drug delivery in association with the tumor microenvironment.

Matrix metalloproteinases (MMPs) are zinc-dependent endopeptidases [2, 3]; among the MMP members, the membrane type 1 (MT1)-MMP/MMP-14 plays a vital role in formation and progression of most form of human tumors [4]. MMP-14 is not or low expressed in majority of normal tissues, but widely expressed and critical to the acquisition of the invasive and metastatic phenotype of prostate, breast, melanoma and ovarian carcinomas [5]; therefore, targeting MMP-14 is an effective approach to cancer therapy.

Tissue inhibitors of metalloproteinases (TIMPs) are endogenous proteins which can inhibit the activity of MMPs. Previous studies have shown that when the balance between TIMPs and MMPs is altered, it could bring about the degradation of the extracellular matrix and induce tumor cell invasion, migration or other receptor-mediated changes [6]. The pleiotropic activities of the four-member TIMP family are perplexing, and hinge upon direct interactions with tumor cells as well as on the sophisticated interactions with other extracellular components to a certain extent [7]. Among the TIMP family members, tissue inhibitor of metalloproteinase 2 (TIMP2) is a distinguished one, because it not only correlates with matrix remodeling and angiogenesis suppressing, but also participates in the process of tumor growth, inflammation and other diseases [8]. Extensive study revealed that TIMP2 possesses the potential as an anticancer agent. TIMP2 can bind to tumor cells in a specific and saturable mode; whereas, the identification of cell surface binding proteins for TIMP2 is complicated by the presence of MMP-14, which is remained poorly characterized [9]. *In vitro* study, MMP-14 is known as an important activator of pro-MMP-2 at the cell surface via the involvement of TIMP2 [10]; for this reason, the interactions among MMP-14, TIMP2 and MMP-2 are of importance in cancer cell invasion and migration. In order to develop a new strategy for targeted therapy, we tried to design and construct fusion proteins on the basis of the MMP-14/TIMP2/MMP-2 tri-molecular interaction model [4]. Briefly, the study takes MMP-14 as the molecular target and employs the TIMP2-based fusion protein as the targeted drug carrier.

Lidamycin (LDM, also called C-1027) is an antitumor antibiotic with extremely potent cytotoxicity. The LDM molecule consists of an active enediyne chromophore (AE, 843 Da) which is responsible for the highly potent bioactivity, and a non-covalently bound apoprotein (LDP, 10, 500 Da) which provides a hydrophobic domain for stabilizing and protecting the former [11]. As reported, AE and LDP can be dissociated and reassembled *in vitro* under certain conditions; notably, the reconstituted LDM displays similar properties to that of natural LDM [12]. LDP and various LDP-containing fusion proteins can be prepared by DNA recombination. Furthermore, enediyne-integrated analogues can be prepared by assembling AE into the engineered LDP-containing fusion proteins.

In the present study, TIMP2-based and LDP-containing fusion proteins, including LDP-TIMP2 and TIMP2-LDP, were generated through the *Pichia pastoris* expression system; and then the enediyne-integrated analogues LDP(AE)-TIMP2 and TIMP2-LDP(AE) were prepared as the above-mentioned procedure, respectively. The study provides evidence that LDP-TIMP2 possesses preferable targeting property than TIMP2-LDP; additionally, the enediyne-integrated analogue LDP(AE)-TIMP2 shows potent antitumor efficacy *in vitro* and *in vivo*.

## RESULTS

### Construction, preparation and characterization of fusion proteins and their enediyne-integrated analogues

**Figure 1:**
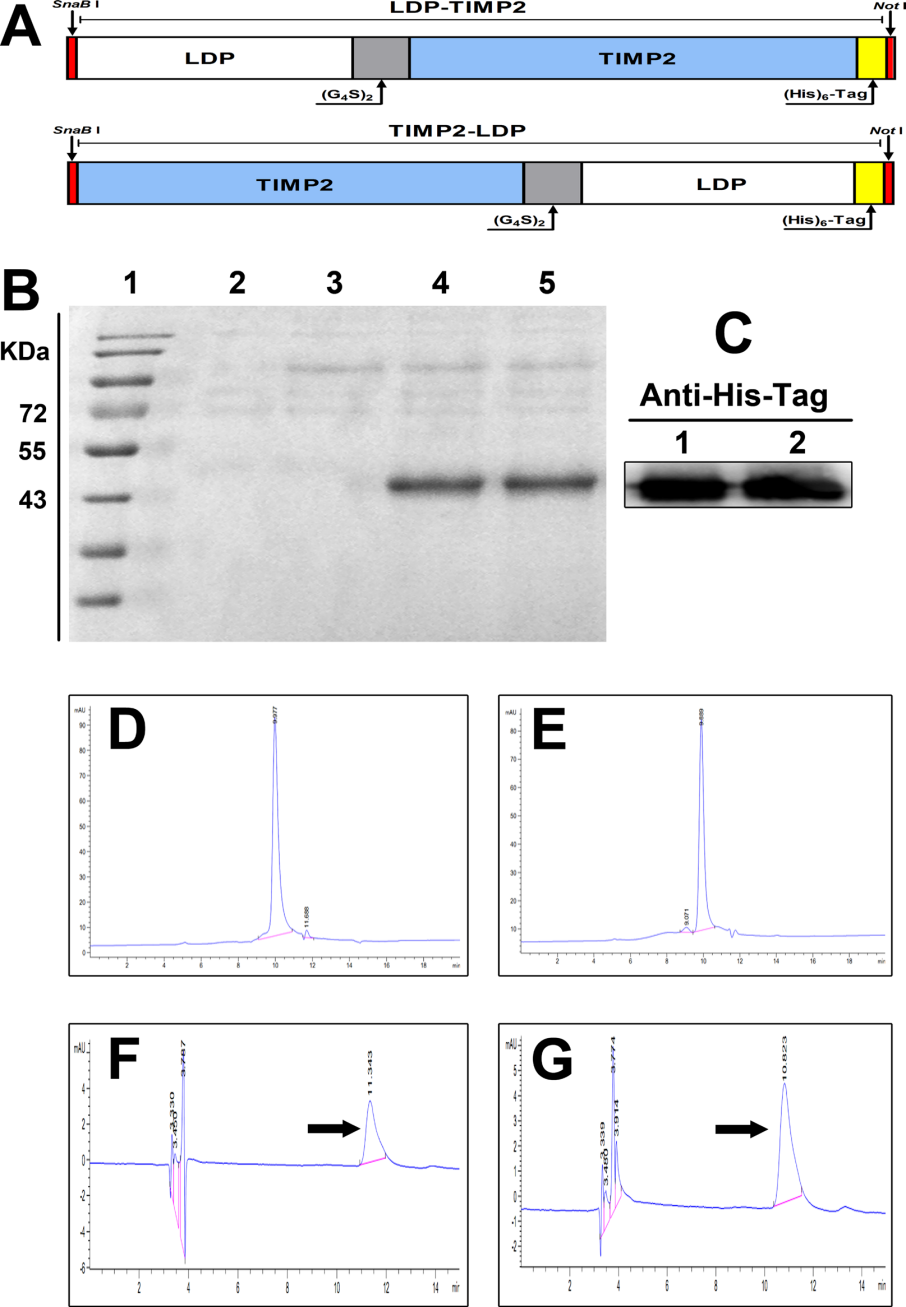
Construction, expression of the fusion proteins, and preparation of their enediyne-integrated analogues **A.** Diagram of *SnaB I*/*Not I* gene fragments encoding for the fusion proteins LDP-TIMP2 (upper row) and TIMP2-LDP (lower row), respectively. **B.** Expression analysis of fusion proteins LDP-TIMP2 and TIMP2-LDP by 12% SDS-PAGE. Lane 1, molecular weight marker; Lane 2, empty vector as a control; Lane 3, without the addition of methanol; Lane 4, expression analysis of LDP-TIMP2; Lane 5, expression analysis of TIMP2-LDP. **C.** Western blotting detection of the fusion proteins LDP-TIMP2 and TIMP2-LDP using mouse anti-His tag monoclonal antibody (1/1000 dilution) and HRP-conjugated goat anti-mouse IgG (1/2000 dilution). **D.** HPLC analysis for the purity of fusion protein LDP-TIMP2. **E.** HPLC analysis for the purity of fusion protein TIMP2-LDP. **F.** Reverse-phase HPLC analysis for the enediyne-integrated fusion protein LDP(AE)-TIMP2 using a Vydac C4 300A column at 340 nm. **G.** Reverse-phase HPLC analysis for the enediyne-integrated fusion protein TIMP2-LDP(AE) using a Vydac C4 300A column at 340 nm.

As shown in Figure 1A, The DNA fragments encoding for fusion proteins LDP-TIMP2 and TIMP2-LDP were obtained by genetic engineering. The primary determination of protein expression strains was analyzed by SDS-PAGE and Western blotting, which were presented at Figure 1B and Figure 1C. The purity of LDP-TIMP2 and TIMP2-LDP was assayed by HPLC and shown in Figure 1D and 1E. Following, the enediyne-integrated fusion proteins were prepared by assembling the active AE molecule of LDM into LDP-TIMP2 and TIMP2-LDP, respectively. The result of reverse-phase HPLC in Figure 1F and 1G showed that the AE molecule was assembled successfully.

### Binding affinity of the fusion proteins *in vitro*

**Figure 2:**
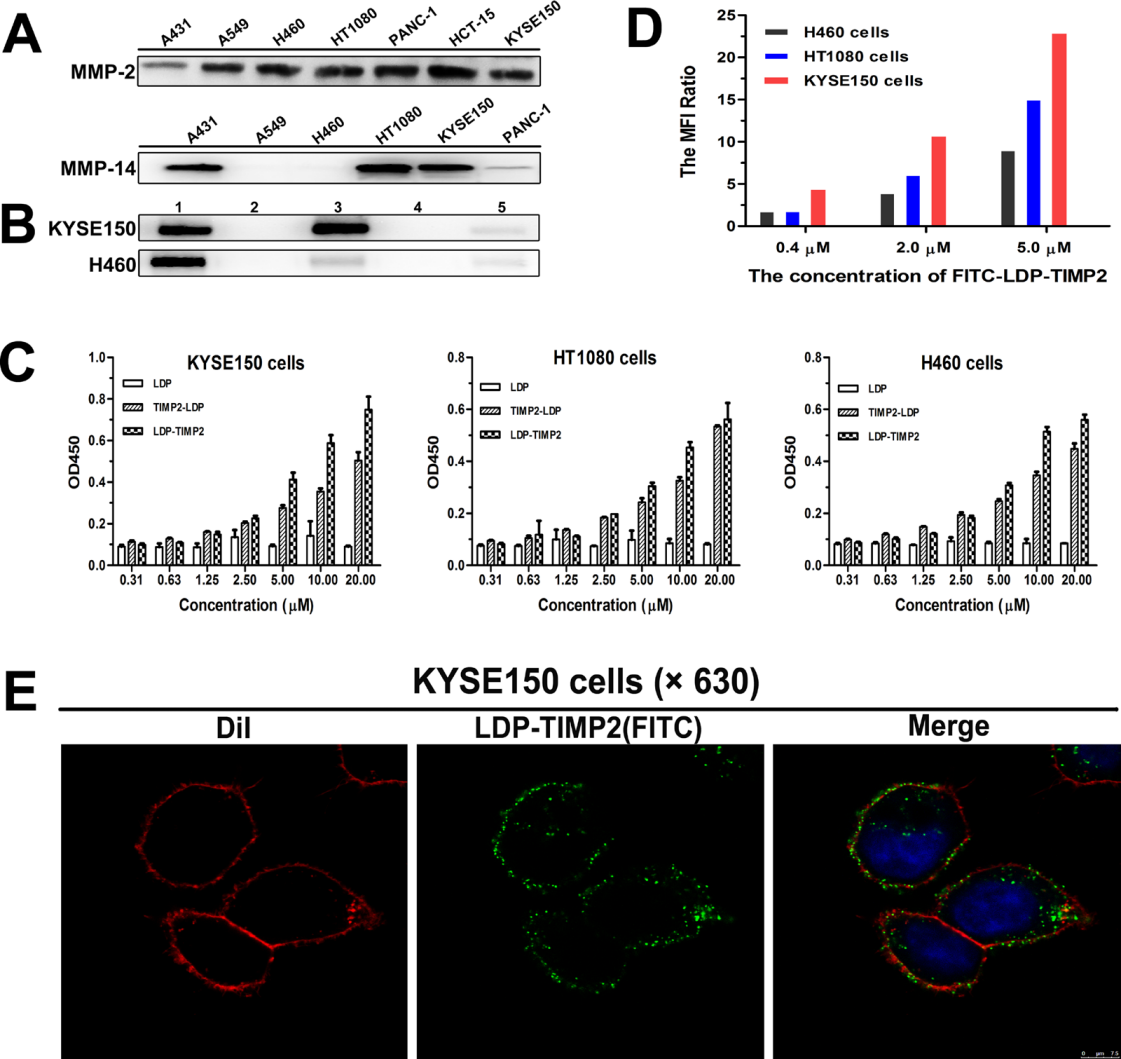
Binding affinity analyses of fusion proteins *in vitro* **A.** Expression levels of MMP-2 (upper row) and MMP-14 (lower row) on different cancer cell lines analyzed by Western blotting. **B.** Co-IP analysis for KYSE150 cells (upper row) and H460 cells (lower row). Total proteins extracted from KYSE150 cells and H460 cells were incubated with LDP-TIMP2 (Lane 1, 3 and 5) and PBS (Lane 2 and 4), respectively. Subsequently, the mixture was incubated with anti-MMP-2 monoclonal antibody (Lane 1 and 2), anti-MMP-14 monoclonal antibody (Lane 3 and 4), or pre-immune serum (Lane 5), separately. The formed complexes were collected with Protein A+G Agarose and analyzed by SDS-PAGE and immunoblotting with anti-His-tag monoclonal antibody. **C.** Binding affinity analyses of three proteins to KYSE150, HT1080 and H460 cells by ELISA. **D.** Binding affinity of various concentrations of FITC-labeled LDP-TIMP2 to KYSE150, HT1080, and H460 cells in FACS analysis (The MFI Ratio = MFI experiment group: MFI control group). **E.** Confocal-based binding and uptake analyses in KYSE150 cells. The images were observed under the LEICA TCS SP5 (×630). The merged image is with DiI staining (red), DAPI staining (blue), and FITC-LDP-TIMP2 (green).

Seven cancer cell lines were analyzed for MMP-2 and MMP-14 expression levels by Western blotting analysis. As shown in Figure 2A, high level expression of MMP-2 was found in six of the tested cell lines, including KYSE150, H460, HT1080 and others. Higher level expression of MMP-14 was detected in three of the six cell lines, namely, KYSE150, HT1080 and A431 cells. The Co-IP results in Figure 2B showed the binding capability of LDP-TIMP2 to both MMP-14 and MMP-2 in KYSE150 cells. In addition, the binding of LDP-TIMP2 to MMP-14 in KYSE150 cells was more intensive than that in H460 cells.

To compare the binding efficiency of LDP-TIMP2 and TIMP2-LDP to various cancer cell lines, KYSE150, HT1080 and H460 cells were examined by ELISA. At the same time, the protein LDP was used as control. As shown in Figure 2C, both of the fusion proteins bound to the three tested cell lines positively. In addition, the binding intensity of LDP-TIMP2 was stronger than that of TIMP2-LDP in KYSE150 cells.

The binding capability of FITC-labeled LDP-TIMP2 to KYSE150, HT1080 and H460 cells was evaluated by FACS analysis (Figure 2D). As shown, the binding and uptake occurred in a concentration-dependent manner in the three tested cell lines; moreover, the intensity was much higher in KYSE150 cells. As presented in Figure 2E, the binding and uptake of LDP-TIMP2 in KYSE150 cells were further confirmed by using laser scanning confocal microscope (LEICA TCS SP5). When FITC-labeled LDP-TIMP2 incubated with KYSE150 cells at 37°C for 1 h, it clearly showed that the fluorescence distributed on cell membrane and in cytoplasm.

### Tissue microarray and immunohistochemistry

**Figure 3:**
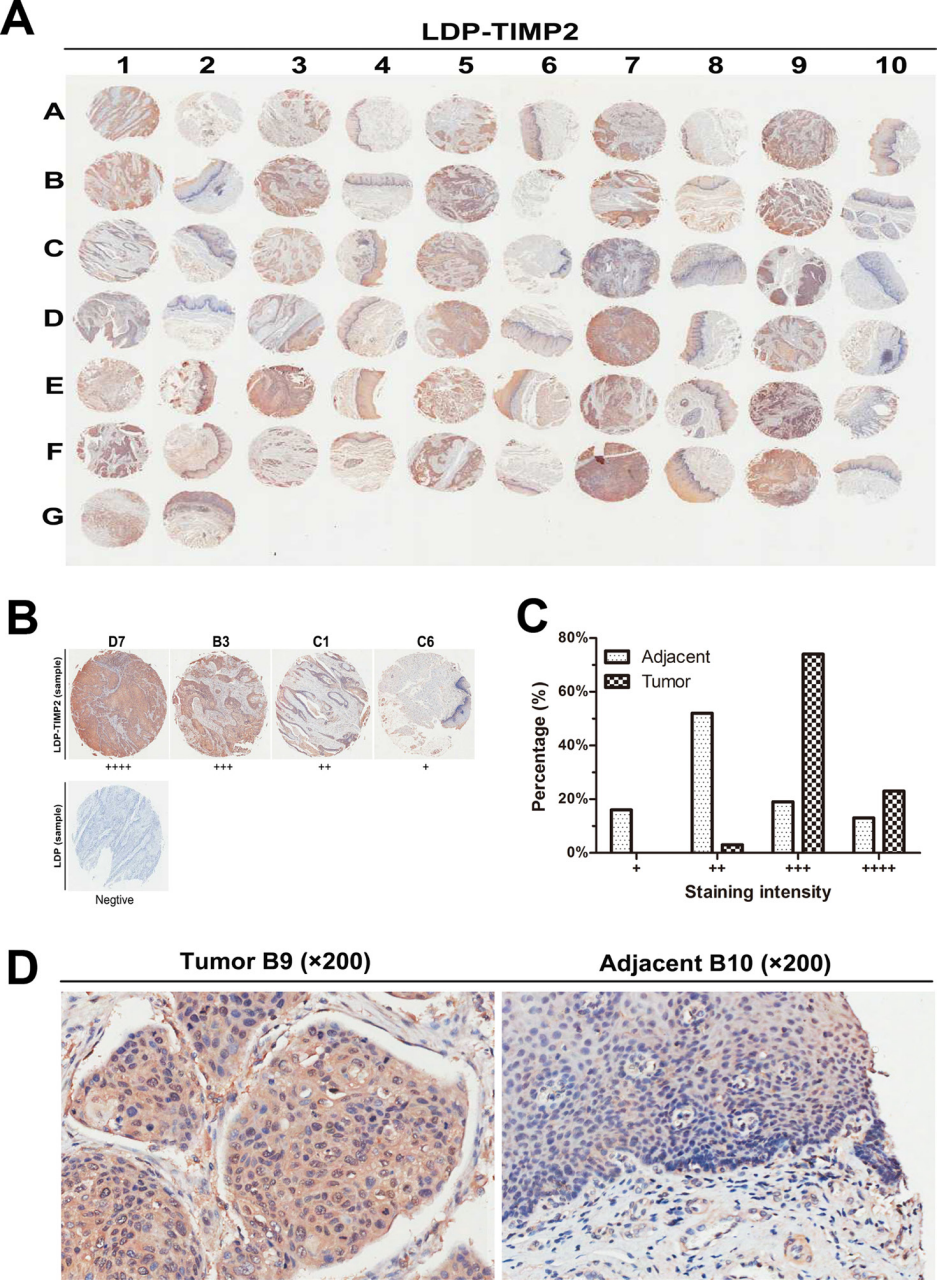
Binding affinity analysis via tissue microarray **A.** Overview of the tissue microarray (LDP-TIMP2); Column 1, 3, 5, 7, 9, samples of esophageal carcinoma tissue, column 2, 4, 6, 8, 10, samples of matched adjacent tissue. **B.** The Grading standards for the positive cases (LDP-TIMP2, ×40) and negative staining case (LDP, ×40) were shown. **C.** Percentages of tumor samples and adjacent samples according to the standards evaluation of the binding of LDP-TIMP2. **D.** Representative cases illustrating binding patterns of LDP-TIMP2 in tumor B9 (left, ×200) and matched adjacent tissue B10 (right, ×200).

On the basis of the above binding affinity assessment *in vitro*, we tested the binding capability of LDP-TIMP2 and LDP to human cancer specimens through tissue microarray of esophageal squamous cell carcinoma and matched adjacent tissues. The scanning pattern image of LDP-TIMP2 was presented in Figure 3A. The evaluation standards were presented in Figure 3B. According to the evaluation standards, the classification of samples was shown in Figure 3C. As shown in Figure 3D, the representative results between tumors and their matched adjacent tissues denoted significant difference. Evidently, it suggests that the fusion protein LDP-TIMP2 binds to tumor tissue preferably as compared with matched adjacent tissue.

### *In vivo* imaging of fusion proteins

**Figure 4:**
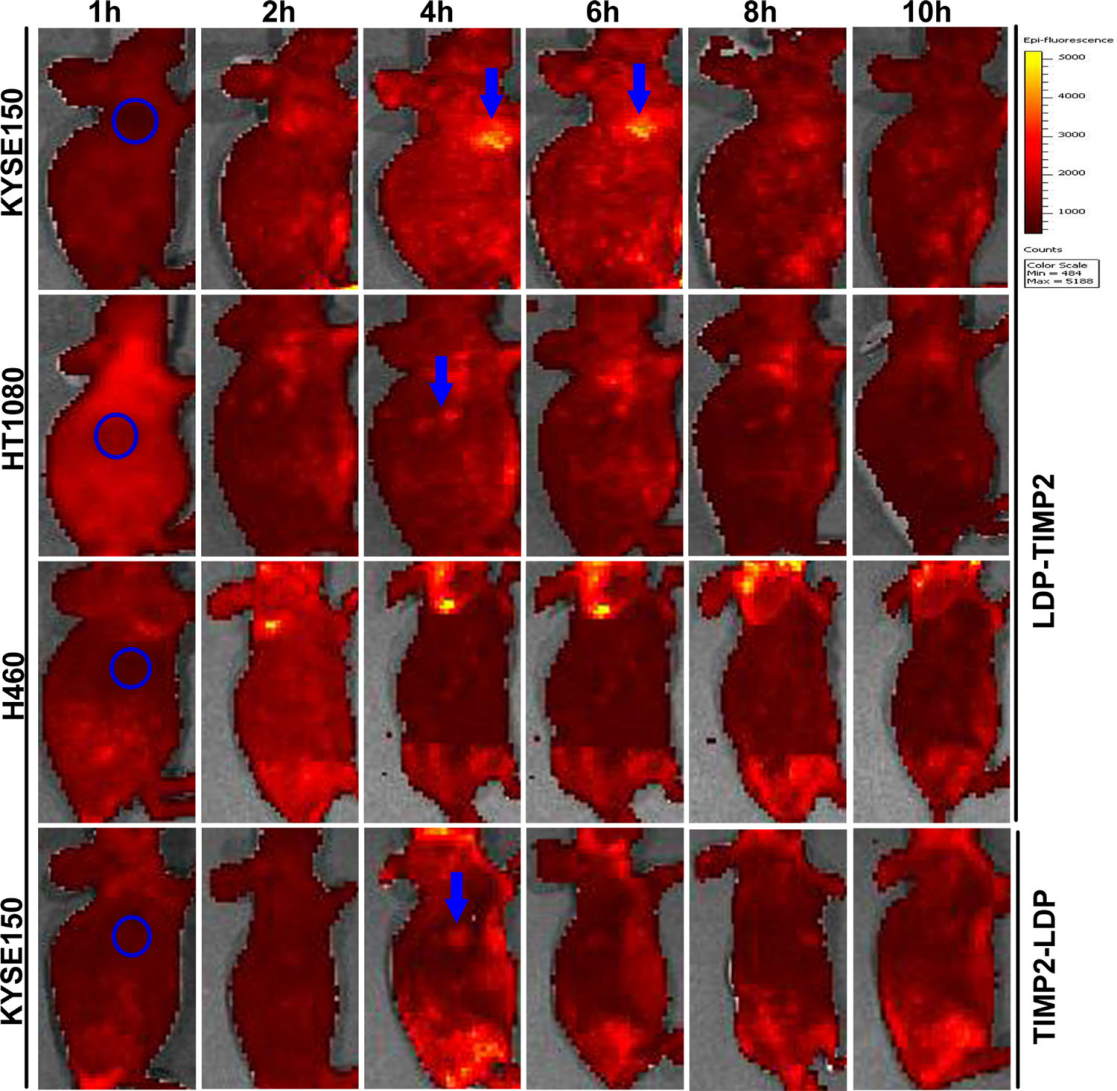
Representative *in vivo* fluorescence images of KYSE150, HT1080 and H460 xenograft-bearing athymic mice at different time points after tail vein injection of FITC-labeled LDP-TIMP2 or TIMP2-LDP The blue circled area indicates the tumor location. The blue arrow shows the targeted site. Color scale represents photons/s/cm^2^/steradian.

*In vivo* imaging of the fusion proteins LDP-TIMP2 and TIMP2-LDP in cancer xenograft-bearing athymic mice are shown in Figure 4. LDP-TIMP2 showed better targeting activity to KYSE150 tumor xenograft than that to HT1080 and H460 tumors, by contrast, TIMP2-LDP showed little accumulation in tumor location. This observation was consistent with *in vitro* results, which further indicated that LDP-TIMP2 was more suitable than TIMP2-LDP as a targeting delivery carrier.

### The anti-angiogenic function of TIMP2-based fusion proteins

**Figure 5:**
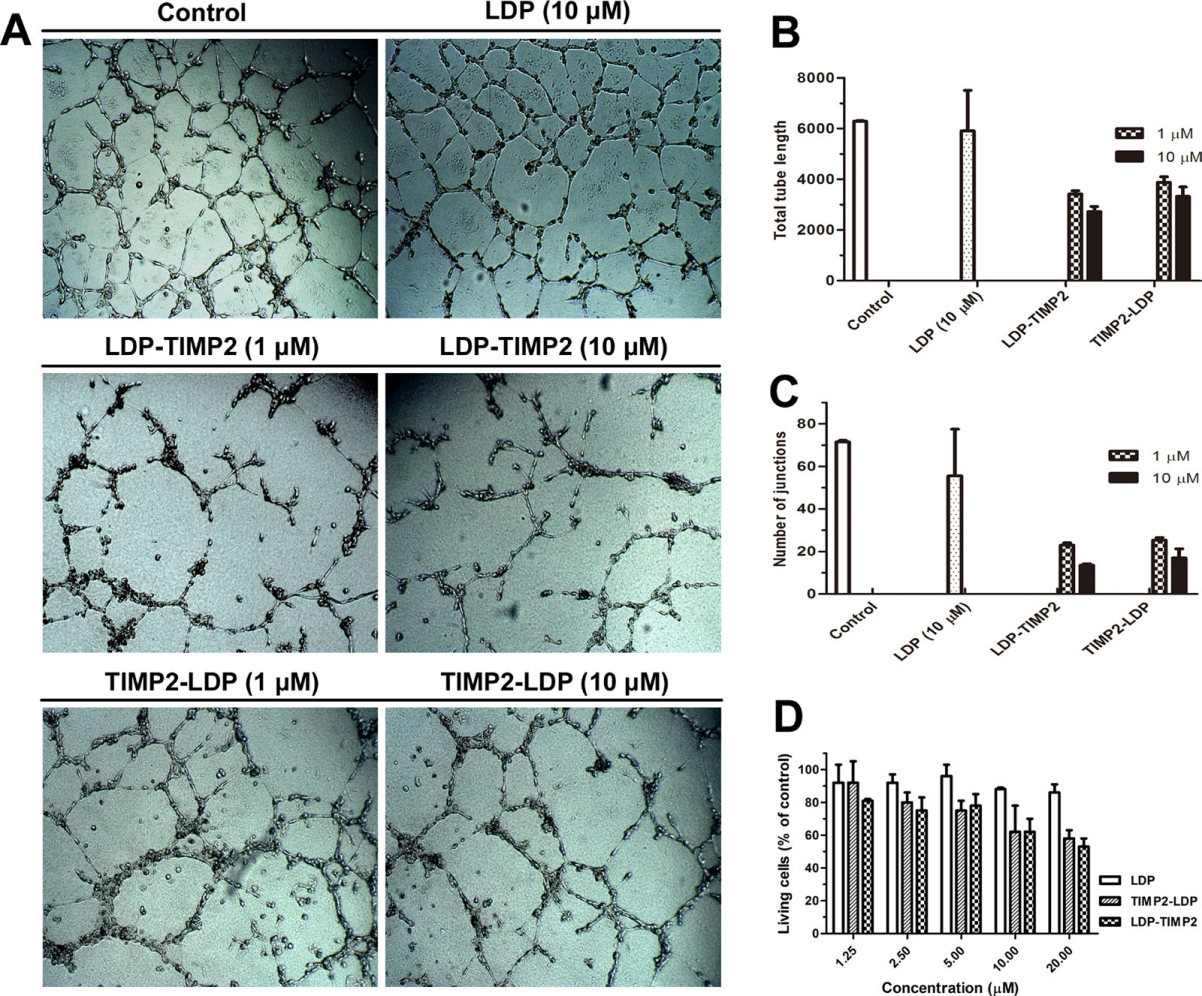
Anti-angiogenic effects of TIMP2-based fusion proteins **A.** Effects of LDP-TIMP2, TIMP2-LDP and LDP on tube formation of HUVEC cells in matrigel membrane matrix (×40). **B.** Change in the total tube length after different treatments. **C.** Change in the number of junctions after different treatments. **D.** Inhibition of HUVEC cell proliferation by different treatments (Determined by Cell Counting Kit-8 assay).

Anti-angiogenesis is considered to be a promising strategy for inhibiting tumor growth and metastasis [13, 14]. As reported, no matter the mechanisms are dependent or independent of MMPs inhibition, TIMP2 can suppress human umbilical vein endothelial cells (HUVEC) proliferation and angiogenesis *in vitro* [15, 16]. In this study, an endothelial tube formation assay was used to evaluate the anti-angiogenic function of TIMP2-based fusion proteins. Determined by total tube length and number of junctions, both LDP-TIMP2 and TIMP2-LDP inhibited tube formation and their efficacy were stronger than that of LDP (Figure 5A and 5C). Moreover, both LDP-TIMP2 and TIMP2-LDP suppressed HUVEC cells proliferation as determined by Cell Counting Kit-8 assay (Figure 5D).

### *In vitro* efficacy of enediyne-integrated fusion proteins

The cytotoxicity of enediyne-integrated fusion proteins LDP(AE)-TIMP2 and TIMP2-LDP(AE) to KYSE150, HT1080, H460 and A549 cells was investigated. For comparison, natural LDM was used as positive control. Determined by MTT assay, both of the TIMP2-based, enediyne-integrated fusion proteins LDP(AE)-TIMP2 and TIMP2-LDP(AE) displayed highly potent cytotoxicity to the tested cancer cell lines. For KYSE150 cells, the IC50 values of LDP(AE)-TIMP2 and TIMP2-LDP(AE) were 4.31 × 10^−11^M and 1.10 × 10^−10^ M, respectively (Table 1).

**Table 1:** Cytotoxicity of the enediyne-integrated fusion proteins to various cancer cell lines (Determined by MTT assay)

Cell line	IC50 (M)		
	LDM	TIMP2-LDP(AE)	LDP(AE)-TIMP2
**KYSE150**	1.28 × 10^−10^	1.10 × 10^−10^	4.31 × 10^−11^
**HT1080**	3.71 × 10^−11^	1.37 × 10^−10^	4.43 × 10^−11^
**H460**	1.48 × 10^−12^	2.06 × 10^−12^	1.81 × 10^−11^
**A549**	1.10 × 10^−11^	2.09 × 10^−11^	4.91 × 10^−12^

### *In vivo* therapeutic efficacy of enediyne-integrated fusion protein LDP(AE)-TIMP2

**Figure 6:**
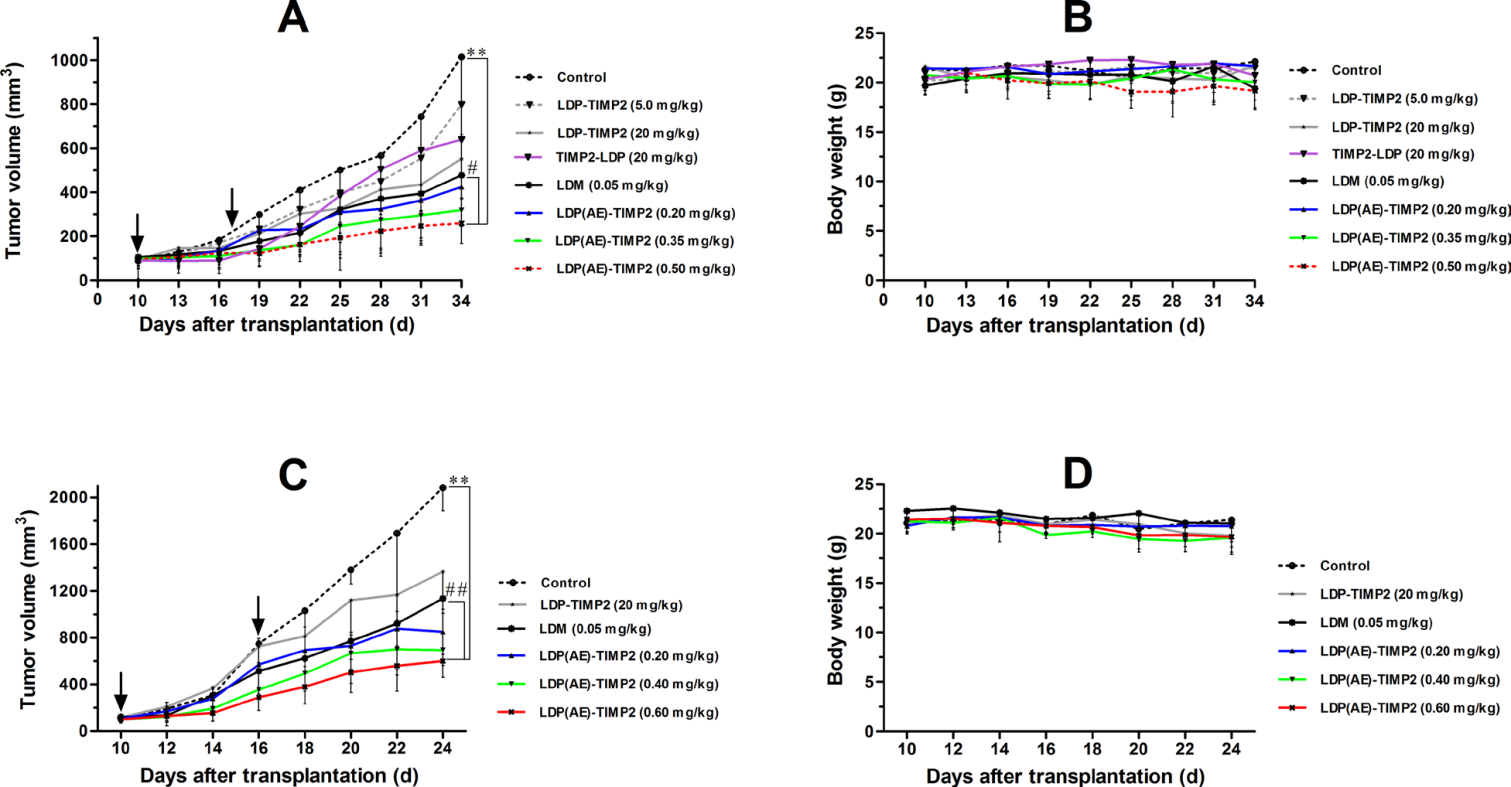
*In vivo* antitumor efficacy of LDP(AE)-TIMP2 on human cancer xenografts in athymic mice The drugs were injected intravenously on days as arrows indicated. **A.** Tumor growing curves of the esophageal carcinoma KYSE150 xenograft (*n* = 6). **, *P* ≤ 0.005, compared with the control; #, *P* ≤ 0.05, compared with the LDM group. **B.** Body weight change of KYSE150 xenograft-bearing mice. **C.** Tumor growing curves of the fibrosarcoma HT1080 xenograft (*n* = 6). **, *P* ≤ 0.005, compared with the control; ##, *P* ≤ 0.005, compared with the LDM group. **D.** Body weight change of HT1080 xenograft-bearing mice.

Human esophageal carcinoma KYSE150 xenograft and human fibrosarcoma HT1080 xenograft in nude mice were used for evaluating *in vivo* antitumor efficacy. Experiment 1 (shown in Figure 6A and 6B) was set for investigating the therapeutic efficacy in KYSE150 xenograft in athymic mice. Tested agents were administered intravenously, once a week, a total of 2 injections. For the control group, mice were injected with physiological saline. As shown in Figure 5A, the enediyne-integrated fusion protein LDP(AE)-TIMP2 markedly inhibited the growth of KYSE150 xenograft. Determined by the end of the experiment, LDP(AE)-TIMP2 at doses of 0.20 mg/kg, 0.35 mg/kg and 0.50 mg/kg inhibited the growth of KYSE150 xenograft by 64%, 76% and 82%, respectively; while LDM inhibited tumor growth by 60%. No deaths and no major body weight changes were found in mice of treated groups (Figure 5B). These results suggested that the TIMP2-based and enediyne-integrated protein LDP(AE)-TIMP2 was highly effective against esophageal squamous carcinoma xenograft.

Experiment 2 was set for evaluation of the therapeutic efficacy in HT1080 xenograft in athymic mice. Tested agents were administered intravenously, a total of 2 injections with a 6 day interval. The control group was treated according to the Experiment 1. As shown in Figure 6C, LDP(AE)-TIMP2 suppressed the growth of HT1080 xenograft by 62%, 70% and 75%, respectively, at doses of 0.20 mg/kg, 0.40 mg/kg and 0.60 mg/kg; while LDM by 49%. There were no significant body weight changes (Figure 6D) and no deaths during the whole observation process.

By histopathological examination, no toxico-pathological changes were found in the heart, lung, liver, small intestine, kidney and femur bone marrow of the athymic mice treated with various dosage levels of LDP(AE)-TIMP2. This indicated that the administered doses of tested agents were well tolerated.

## DISCUSSION

Currently, matrix metalloproteinases (MMPs) are known to be one of the most prominent family of proteinases associated with tumorigenesis; particularly, its family member MMP-14 causes much attention as a promising drug target for its membrane-anchored characteristic and the effects in promoting various cancers progression [17]. As reported, MMP-14 plays a pivotal role in conferring tumor cells with the capability to degrade extracellular matrix ingredients, correlating with tumor invasion and metastasis; MMP-14 is also involved in regulating the release of angiogenic factors in tumor microenvironment and promoting blood vessel formation associated with tumor growth [18, 19]. Recent studies indicated that higher expression of MMP-14 is related to poorer prognosis and shorter survival time in patients with some types of cancer. Studies on the mechanism of activation have shown that MMP-14 plays a role as the receptor for TIMP2 [20], while the N-terminal domain of TIMP2 can bind with the catalytic domain of MMP-14, as well as MMP2, resulting in the formation of a MMP-14/TIMP2/MMP-2 tri-molecular complex. This provides evidence that TIMP2 can bind to MMP-14 in a unique manner, accordingly, TIMP2 may be able to serve as a MMP-14 directed carrier for targeted drug delivery.

In tumor microenvironment, the relation of cancer cells and the stroma is highly complicated and it keeps changing over time. Collaborative interactions among cancer cells with cancer-associated fibroblasts (CAFs), vascular system as well as a dynamic network of the extracellular matrix (ECM), might promote tumor formation, unlimited progression or eventual metastasis [21]. As shown, normal tissue fibroblasts primarily suppress tumor formation; however, CAFs can probably involve in tumorigenesis [22]. Moreover, uneven or defective vascular system can generate distinct TME, promoting tumor heterogeneity and finally affecting the pre-clinical or clinical therapeutic effects. Besides tumor cells, different types of stromal cells produce and release a number of specific MMPs and their natural inhibitors, and the delicate balance between MMPs and TIMPs potentially determines the progression of tumors. Accumulating studies have provided firm supports for the important roles of TIMPs in the TME. Among the four members of human TIMP family, TIMP2 has been investigated as a promising antitumor agent for its essential functions in tissue remodeling, tumor growth and angiogenesis inhibition [23]. TIMP2 can increase cell-cell adhesion, effectively inhibiting tumor growth, migration, and epithelial to mesenchymal transition (EMT). TIMP2 can reduce the phosphorylation of ERK and AKT, subsequently, suppress endothelial growth and angiogenesis [24]. TIMP2 can also bind to the integrin-α_3_β_1_ receptor [25], which causes the signaling cascades, eventually lead to the hypo-phosphorylation of VEGFR-2 and the up-regulation of the anti-migration factor, RECK.

Targeted therapy encompasses a wide variety of strategies. Especially, entire monoclonal antibody or antibody fragment-based drugs have been found to be effective in this area [26, 27]; furthermore, antibody and antibody-drug conjugate (ADC) therapeutics have been applied for cancer treatment [28-30]. In recent years, several MMP-14-directed antibodies have been developed and their reactivity with the target have been reported [4, 5, 31]. In addition, the underlying molecular mechanisms of antibody action have been investigated [32, 33], however, antibody-drug conjugate and fusion protein targeting MMP-14 have not yet been reported. Based upon these, it is interesting to seek an alternative and effective way for targeting MMP-14, therefore, we put forward a new strategy for MMP-14 targeted therapy that uses TIMP2 as the binding molecule on the basis of its specific interaction with MMP-14 to prepare relevant drug conjugates or fusion proteins.

LDM is known for its extremely potent cytotoxicity against cultured cancer cells, compared in terms of IC50 values, the cytotoxicity is over 1, 000 times more potent than that of doxorubicin. In our laboratory, we have designed a variety of antibody or ligand-based targeted drugs, for instance, EGFR/HER2 bispecific and enediyne-energized fusion protein Ec-LDP-Hr-AE [34], gelatinase targeting diabody-based fusion protein dFv-LDP-AE [35], as well as a novel polymer-protein conjugate, Dex-rLDP-AE [36]; accordingly, obtained preferable targeted attributes and significant therapeutic efficacy in nude mice xenograft models. In this study, we constructed a TIMP2-based and enediyne-integrated fusion protein LDP(AE)-TIMP2 that targets MMP-14; actually, it is a TIMP2-drug conjugate (TDC) in nature, close to ADC in many aspects. For the purpose of avoiding the renaturation troubles of inclusion bodies in *Escherichia coli* expression system, we employed *Pichia pastoris* secretory expression system to produce the fusion protein, further guaranteeing the biologic activity of each component. In summary, we consider that the TIMP2-based and enediyne-integrated fusion protein LDP(AE)-TIMP2 could be a promising agent in cancer targeted therapy; without doubt, further validations of the drug are also needed.

## MATERIALS AND METHODS

### Cell lines and culture

The following cell lines including human epidermoid carcinoma A431, human lung carcinoma A549 and H460, human esophageal carcinoma KYSE150, human fibrosarcoma HT1080, and human colorectal adenocarcinoma HCT-15 were cultured in modified RPMI-1640 (Hyclone; Thermo Fisher Scientific) supplemented with 10% (v/v) of heat-inactivated fetal bovine serum (FBS, Gibco; Life Technologies), penicillin G (100 U/mL), and streptomycin (100 μg/mL). The human pancreatic carcinoma cell line PANC-1 was cultured in Dulbecco's modified Eagle medium (Hyclone; Thermo Fisher Scientific) supplemented with the same ingredients. Human umbilical vein endothelial cells (HUVEC) were cultured in VascuLife basal medium (LIFELINE Cell Technology) supplemented with LifeFactors^R^. All cell lines were cultured in an incubator, maintained at 37°C with 5% CO_2_.

### Construction of expression vectors

As shown in Figure 1A, the full gene fragments of the fusion proteins LDP-TIMP2 and TIMP2-LDP mainly consist of the gene encoding TIMP2 (194 amino acids), apoprotein LDP (110 amino acids) and the linker peptide (GlyGlyGlyGlySer)_2_. After molecular biology process, the resultant 942-bp fragments were digested by *SnaB I*/*Not I*, inserted into expression vector to generate expression plasmids pHBM-LDP-TIMP2 and pHBM-TIMP2-LDP. DNA sequencing determination was accomplished by the method provided by Invitrogen Corp.

### Expression, purification of fusion proteins and preparation of their enediyne-integrated analogues

For *Pichia pastoris* strain GS115 transformation, the expression plasmids pHBM-LDP-TIMP2 and pHBM-TIMP2-LDP were transformed into cells by electroporation using 2 mm gap cuvettes. After 3-5 days culture, PCR was performed to determine the genes being integrated into yeast chromosome. Glycerol stock of GS115-pHBM-LDP-TIMP2 and GS115-pHBM-TIMP2-LDP were inoculated into 30 mL of BMGY medium (1% Yeast extract, 2% Bacto peptone, 1% Glycerol, 1.34% YNB, 4 × 10^−5^ % Biotin, with 100 mM Potassium phosphate buffer, pH 6.0), and incubated in Erlenmeyer flasks on a rotary shaker for 36 h (30°C, 280 rpm). The cells were pelleted by centrifugation at 3, 000 × g for 5 min and resuspended in BMMY medium (1% Yeast extract, 2% Bacto peptone, 1% Methanol, 1.34% YNB, 4 × 10^−5^ % Biotin, with 100 mM Potassium phosphate buffer, pH 6.0). The culture was maintained for another 96 h with the addition of 1% (v/v) pure methanol at every 24 h to sustain the induction conditions. Subsequently, the culture was harvested by centrifugation (3, 000 × g for 10 min) at room temperature (RT), and the supernatant collected both from pre-induced and induced cultures was analyzed for the expression of the proteins by SDS-PAGE under denaturing conditions. Cell growth and recombinant proteins production during the induction phase were optimized in shake flasks by analyzing the effect of pH, temperature, induction time as well as the addition of methanol [37, 38]. The fusion proteins were purified by affinity chromatography (His Trap HP, GE Healthcare) according to the operation manual. The protein concentration was determined by the BCA protein assay kit (Thermo Fisher Scientific, USA).

The AE of LDM was separated by C4 column (GE Healthcare) with a 22% acetonitrile in 0.05% trifluoroactic acid mobile phase. For preparation of enediyne-integrated fusion proteins LDP(AE)-TIMP2 and TIMP2-LDP(AE), the AE-containing solution was added to LDP-TIMP2/PBS (10 mM pH7.4) or TIMP2-LDP/PBS, respectively, with the molecular ratio of 3:1, and incubated at 4°C for more than 12 h while rocking. Finally, free AE was removed with a Sephadex G-75 column (GE Healthcare). The resultant enediyne-integrated fusion proteins named LDP(AE)-TIMP2 and TIMP2-LDP(AE) were confirmed by reverse-phase HPLC using a Vydac C4 300A column (Grace), respectively. Absorbance at 340 nm was measured accordingly.

### Western blotting and co-immunoprecipitation analyses

Cell lysates were collected for analysis of MMP-2 and MMP-14 protein levels. After the cultured cells reached approximately 85% confluence, the culture supernatant was removed and the cells were washed three times with PBS. Then the total cellular protein was obtained using an ice-cold high efficiency RIPA tissue/cell lysis buffer supplemented with 1% (v/v) of protease inhibitor at 4°C for 20 min (Beijing Solarbio Science & Technology Co., Ltd). After centrifugation at 4°C (15, 000 × g for 15 min), the supernatant was harvested and the total protein was quantified. The same amount of protein for each cell sample (30 μg) was separated by 12% SDS-PAGE and transferred onto a PVDF membrane, then blocked with 5% skim milk for 2 h, incubated with respective primary antibody (MMP-2 antibody, 1/1000 dilution, Cell Signaling Technology; MMP-14 antibody, 1/1000 dilution, Abcam) at 4°C overnight and with HRP-conjugated secondary antibody (1/2500 dilution, Zhongshan Golden Bridge Biotechnology) at RT for 2 h. The specific bands were visualized using an enhanced chemiluminescence kit (Merck Millipore, USA). At least three independent experiments were carried out in Western blotting analysis.

For co-immunoprecipitation assay, KYSE150 and H460 cells were pretreated as those in Western blotting. Thereafter, 200 μg of protein samples extracting from cell lysates were incubated with 50 μg LDP-TIMP2 protein at 4°C for 2 h under rotation, followed by overnight incubation with 0.3 μg anti-MMP-2 or anti-MMP-14 antibody at 4°C under rotation. Subsequently, 40 μL of Protein A+G Agarose (Beyotime, China) were added at 4°C for 2 h under rotation. The pellets were collected by centrifugation (1, 000 × g for 5 min) and washed with PBS 5 min each for five times. Then, the cells were resuspended in 40 μL of electrophoresis loading buffer and boiled for 10 min. Finally, SDS-PAGE and Western blotting analysis were performed as described above. The mouse anti-His tag monoclonal antibody (1/2000 dilution; Abmart Biotechnology) and HRP-conjugated goat anti-mouse IgG were used for detecting LDP-TIMP2 protein [34].

### Binding affinity analyses of fusion proteins

Enzyme-linked immunosorbent assay (ELISA) was used for measuring the binding efficiency of LDP, TIMP2-LDP and LDP-TIMP2 to tumor cells. Cells (1 × 10^4^ per well) were seeded in 96-well plates and incubated at 37°C with 5% CO_2_ for 24 h. After fixed with 4% formaldehyde at 4°C for 30 min and blocked with 1% BSA/PBS at 4°C overnight, the cells were incubated with serial concentrations of proteins at 37°C for 2 h, and then, mouse anti-His tag monoclonal antibody (1/2000 dilution) at 37°C for 2 h and horseradish peroxidase-conjugated goat anti-mouse IgG (1/2500 dilution) at 37°C for 2 h, respectively. Subsequently, 3, 3′, 5, 5′-tetramethylbenzidine (Soluble TMB Substrate Solution; Tiangen Biotechnology, Beijing, China) was added at RT for 20 min, and followed by the addition of stop solution (2 M H_2_SO_4_). The absorbance at 450 nm was measured using a microplate reader (Multiskan MK3; Thermo Fisher Scientific, MA, USA). All assays were carried out in triplicate.

For flow cytometry analysis, cell suspensions (5 × 10^5^ cells/sample) were incubated with various concentrations of FITC-labeled LDP-TIMP2 at RT for 2 h with slow shaking. Cells were harvested by centrifugation (1, 000 rpm for 5 min). After three times of PBS washing, cells were resuspended in 500 μL of PBS, and analyzed using a flow cytometer (BD FACS Calibur).

The laser scanning confocal microscope-based analysis was also designed to examine binding and uptake ability of KYSE150 cells to LDP-TIMP2 [39].

### Tissue microarray and immunohistochemistry

The tissue microarray (TMA) chips containing a total of 31 pairs of esophageal squamous cell carcinoma and matched adjacent tissues (OD-CT-DgEso03-002) were provided and processed by Shanghai Outdo Biotech Co., Ltd. (Shanghai, China). Briefly, the main experimental procedure is as follows: sections were proceeded as dewaxing, microwave antigen retrieval, endogenous peroxidase blocking, and then incubated with the primary antibody (fusion protein LDP-TIMP2 or protein LDP) overnight, and the second antibody (His-Tag mAb/HRP Conjugated) for 0.5 h, respectively. Finally, the specimens were determined with DAB detection and hematoxylin staining.

### *In vivo* imaging of fluorescein-labeled proteins

The tumor-targeting ability of LDP-TIMP2 and TIMP2-LDP was investigated using KYSE150, HT1080 and H460 xenografts in athymic mice. When the solid tumors reached a volume of about 100-200 mm^3^, FITC-labeled LDP-TIMP2 (or TIMP2-LDP) was injected into the tail veins of mice (*n* = 3) at a dosage of 30 mg/kg. Following, the mice were anesthetized by isofluorane at a series of time intervals and placed in the imaging chamber of an IVIS-200 system (Xenogen, Alameda, CA, USA) for observation. The images were also processed with imaging system software.

### *In vitro* anti-angiogenic analyses

Tube formation assay was performed as the following protocol [40, 41]. Briefly, matrigel membrane matrix (Vigorous Biotechnology Beijing Co., Ltd) was thawed at 4°C overnight. A 48-well plate and 200 μL pipette tips were also placed on ice during the entire experiment. The plate was coated with 150 uL of matrigel per well and incubated at 37°C for 4 h to allow gelation to occur. Then, HUVEC cells (3 × 10^4^ per well) were plated to the top of the gel in the presence of the LDP, different concentrations of LDP-TIMP2 and TIMP2-LDP, respectively. Tube formation was observed after 18 h and photographed by using an inverted microscope. For comparison of the effect of the tested agents, tube length and number of junctions were determined using Image J software. Each treatment was performed in triplicate.

### Cell viability assay

For the MTT assay, tumor cells were trypsinized and seeded at a density of 4, 000 per well in a 96-well plate, and incubated at 37°C with 5% CO_2_ for 12 h. Then different concentrations of drugs were added in triplicate, respectively. After 48 h, cells were incubated with MTT solution (20 μL, 5 mg/mL) for an additional 4 h. Following, the culture supernatant was removed, 150 μL of dimethyl sulfoxide (DMSO) were added to each well and agitated for 10 min. The absorbance (A) was recorded at 570 nm with a microplate reader (Thermo Fisher Scientific, USA). Untreated cells served as control. The relative cell viability (%) compared with the control was calculated as the following formula: cell viability (%) = [(A_sample_–A_blank_)/(A_control_–A_blank_)] × 100%. The IC50 represented the drug concentration resulting in 50% growth inhibition.

For the Cell Counting Kit-8 (CCK-8, Dojindo, Japan) assay. Briefly, human umbilical vein endothelial cells (HUVEC) were seeded at 3, 000 cells/well into a 96-well plate, and incubated at 37°C with 5% CO_2_ overnight. Then, the culture supernatant was removed, fresh medium as well as various treatments were added in triplicate, respectively. After 48 h incubation, the culture supernatant was removed, 100 μL of fresh medium containing 10% CCK-8 reagent was added and incubated for 4 h. The absorbance was measured at 450 nm using a microplate reader. Untreated cells served as control. The relative cell viability compared with the control was calculated as the formula in MTT assay.

### Animal models for therapeutic efficacy

KYSE150 and HT1080 xenograft models were used for evaluation of *in vivo* antitumor efficacy. Female athymic nude mice (BALB/c, *nu/nu*, 18-22 g) were purchased from the Institute for Experimental Animals, Chinese Academy of Medical Sciences & Peking Union Medical College. All experimental protocols were in accordance with the regulations of Good Laboratory Practice for non-clinical laboratory studies of drugs issued by the National Scientific and Technologic Committee of People’ Republic of China. The 6- to 8-week-old athymic mice were inoculated subcutaneously (s. c.) with exponentially growing human esophageal carcinoma KYSE150 cells (2 × 10^6^ cells per mouse) and human fibrosarcoma HT1080 cells (2 × 10^6^ cells per mouse), respectively.

After about 2 weeks, the grown tumors were excised aseptically. After removing the necrotic parts, the semi-transparent tumor mass was cut into pieces about 2 mm^3^ in size, and transplanted subcutaneously using a trocar separately into the right flanks of mice. When tumors reached about 100 mm^3^ in size, mice were randomly divided into several groups (*n* = 6 per group) and treated with LDP-TIMP2, TIMP2-LDP, natural LDM, or different dosages of LDP(AE)-TIMP2, respectively. The tested agents were injected intravenously through the lateral tail vein, a total of 2 injections with a 7 or 6 day interval. One group of mice was given physiological saline, serving as control. During the experiment period, tumor sizes were measured with a caliper, and tumor volumes were calculated using the formula: V (mm^3^) = 0.5 × *a* × *b^2^*, where *a*, and *b* indicated the long and the perpendicular short diameters of the tumor, respectively. The inhibition rate of tumor growth was calculated as: 100 × {1-[(tumor volume _final_-tumor volume _initial_ for the treated group)/(tumor volume _final_-tumor volume _initial_ for the control group)]}. At the end of the experiment, the mice were euthanized. Specimens of the tumor and various organs were taken and fixed in 10% formalin. Then the paraffin-embedded specimens were cut into sections of 5 μm in thickness and stained with hematoxylin and eosin (H & E). Histopathological changes were observed with the Leica microscope.

### Statistical analysis

All of the experimental data were presented as the mean ± SD. Statistical analysis was performed with Graphpad Prism 5 software. One-way ANOVA followed by Student's *t*-test was used to compare the effect of treatment with the control, and *P* values less than 0.05 were defined to be statistically significant.
